# Values of lymphocyte-related ratios in predicting the clinical outcome of acute ischemic stroke patients receiving intravenous thrombolysis based on different etiologies

**DOI:** 10.3389/fneur.2025.1542889

**Published:** 2025-05-08

**Authors:** Yongyu Li, Keyang Chen, Lu Wang, Linhu Zhao, Chunyan Lei, Yu Gu, Xiaoyan Zhu, Qionghua Deng

**Affiliations:** The First Department of Neurology, First Affiliated Hospital of Kunming Medical University, Kunming, Yunnan, China

**Keywords:** stroke, etiology, thrombolysis, neutrophil, lymphocyte, outcome

## Abstract

**Background:**

While neutrophil-to-lymphocyte ratio (NLR), lymphocyte-to-monocyte ratio (LMR), and platelet-to-lymphocyte ratio (PLR) have been associated with acute ischemic stroke (AIS) outcomes, their differential predictive value across etiological subtypes (TOAST classification) in thrombolysis-treated patients remains underexplored.

**Methods:**

In this retrospective cohort study, we analyzed 381 AIS patients receiving intravenous thrombolysis. Hematological indices were calculated from pre-thrombolysis. Using multivariable logistic regression adjusted for age, NIHSS, and comorbidities, we assessed associations between baseline ratios and 90-day unfavorable outcomes (mRS 3–6). Receiver operating characteristic (ROC) analysis was used to determine optimal cutoffs stratified by TOAST subtypes.

**Results:**

A total of 381 patients were included in the study. NLR showed superior predictive performance: large-artery atherosclerosis: AUC = 0.702 (aOR = 1.35, 95%CI = 1.14–1.61, *p* = 0.001), small-artery occlusion: AUC = 0.750 (aOR = 1.51, 95%CI = 1.08–2.10, *p* = 0.015), cardioembolic stroke: AUC = 0.679 (aOR = 1.82, 95%CI = 1.07–3.10, *p* = 0.028). LMR showed predictive value only in large-artery atherosclerosis (AUC = 0.632, *p* = 0.004). Optimal NLR cutoffs: 3.19 (large-artery), 3.94 (small-artery), 3.17 (cardioembolic stroke).

**Conclusion:**

NLR emerged as a robust, subtype-specific predictor of post-thrombolysis outcomes, particularly in atherosclerotic stroke variants. These findings supported NLR’s clinical utility for risk stratification in thrombolysis-eligible AIS patients.

## Introduction

Stroke was the second leading cause of mortality globally, with acute ischemic stroke (AIS) representing up to 84% of all stroke types (1). AIS resulted in a high incidence of disability, with a prevalence of 20 to 25%, and a mortality rate of 10% (2). These figures impose substantial burdens on society and families. The prognosis of each type of AIS was associated with underlying etiology. The current internationally recognized classification system for AIS based on etiology was derived from a multicenter clinical trial called the Trial of Org 10,172 in Acute Stroke Treatment (TOAST) (3).

A meta-analysis of the literature confirmed that intravenous thrombolysis was the most effective treatment for AIS, which significantly reduced disability and mortality rates (4). Alteplase (rt-PA) has been demonstrated to be an efficacious and widely utilized thrombolytic agent. However, administration of rt-PA is within 4.5 h of symptom onset (5), and its efficacy varies according to the etiology of AIS (4, 6). The rapid and objective identification of ineffective thrombolysis or potential side effects is of the utmost importance for the long-term quality of life of patients.

Animal studies indicate that humoral factors released by dying neurons may trigger inflammation in the injured cerebral area, which may result in the recruitment of peripheral immune cells, including neutrophils, lymphocytes, and monocytes, into the infarcted tissue. This may exacerbate neuronal damage and result in disruption of the blood–brain barrier (7, 8). Neutrophils are the initial inflammatory cells to respond following the onset of AIS, with an increase occurring within 30 min of the initial event, followed by monocytes and lymphocytes (9). These inflammatory responses product a considerable number of inflammatory factors, including interleukins, nitric oxide, interferon gamma, and tumor necrosis factor-alpha (8). The elevated levels of inflammatory factors may induce oxidative stress reactions, which can damage brain tissue cells and result in neurological deficits. The level of platelet monocyte aggregation in patients with a stroke is markedly elevated (10). Platelets, together with these inflammatory cells, participate in the inflammatory development process of cerebral infarction, which lead to microvascular occlusion and exacerbate brain damage (11).

Previous study indicated that higher circulating white cell counts and neutrophil to lymphocyte ratio (NLR) were associated with symptomatic intracerebral hemorrhage (sICH) and poor prognosis in AIS patients receiving intravenous thrombolysis. In addition, some studies had also shown that the NLR, lymphocyte to monocyte ratio (LMR), and platelet to lymphocyte ratio (PLR) at admission were associated with neurological deterioration during hospitalization in AIS patients receiving intravenous thrombolysis treatment (12–14). A recent meta-analysis confirmed a higher level of NLR was associated with adverse outcomes 3 months after thrombolytic therapy in AIS patients (15).

NLR, LMR, and PLR had become effective indicators for evaluating the prognosis of AIS. However, the predictive ability of these inflammatory cell ratios for the prognosis of AIS patients with different etiologies receiving thrombolytic therapy was not clear. This study provided the first direct comparison of NLR, LMR and PLR in functional outcome prediction using standardized modified Rankin Scale (mRS) scores. Values exceeding these cutoffs may justify intensified monitoring or adjuvant anti-inflammatory therapies in specific TOAST subtypes, particularly when combined with higher NIHSS scores or atrial fibrillation.

## Methods

### Study population

This study was a single-center retrospective cohort study. Patients within 4.5 h of AIS onset were enrolled after being admitted to the First Affiliated Hospital of Kunming Medical University, Kunming, China from September 2018 to September 2023, who received intravenous rt-PA treatment. The study protocol was approved by the Scientific Research Department of the First Affiliated Hospital of Kunming Medical University and was designed in accordance with local ethics criteria for human research.

Inclusion criteria: (1) Age ≥18 years old; (2) Onset to treatment time within 4.5 h; (3) Patients receiving rt-PA intravenous treatment. Exclusion criteria: (1) Patients with inflammatory lesions or infectious diseases before admission. The diagnostic criteria for infection require meeting at least two of the following four parameters: (a) Temperature >38°C or <36°C; (b) Heart rate >90 beats per minute; (c) Respiratory rate >20 breaths per minute or hyperventilation (PaCO₂ < 32 mmHg); (d) White blood cell count >12 × 10^9^/L or <4 × 10^9^/L (or immature granulocytes >10%) (16). (2) Patients with absolute contraindications to intravenous thrombolytic therapy; (3) Patients who had not undergone CT or MRI examinations after admission; (4) Those who were unable to get data.

### Data collection

At admission, demographic characteristics, vascular risk factors, past medical history, vital signs, laboratory indicators, thrombolytic therapy-related indicators and imaging indicators before thrombolysis were obtained. National Institutes of Health Stroke Scale (NIHSS) was assessed before thrombolysis. All patients underwent brain Computed Tomography (CT) or Magnetic Resonance Imaging (MRI) scan to exclude intracranial hemorrhage at admission and then received thrombolytic therapy. All patients underwent multimodal CT, MRI, pulmonary CT, carotid ultrasound and echocardiography evaluation to assess etiology, and infarct location.

The etiology classification of all included patients was evaluated by two professional neurologists. All patients underwent standardized etiological workup per the TOAST criteria. Specifically, suspected CE strokes received: ≥24-h cardiac telemetry; transthoracic echocardiography when thrombus was suspected. According to the TOAST criteria to determine the etiology classification of AIS (3). TOAST categorized AIS into five subtypes: large-artery atherosclerosis, cardioembolism, small-vessel occlusion, other determined etiology, and undetermined etiology, and each subtype had specific diagnostic criteria (3).

### Treatment method

Intravenous rt-PA treatment (dosage: 0.9 mg per kg body weight, maximum dose of 90 mg, 10% of the dose given at the first 1 min, the remaining 90% administered as a continuous intravenous infusion within an hour), during thrombolysis and 24 h after intravenous thrombolysis, and no antiplatelet therapy within 24 h after intravenous thrombolysis treatment.

### Neuroimaging assessment

According to the European Cooperative Acute Stroke Study (ECASS) II criteria (17), sICH was defined as any intracranial bleeding within 7 days after thrombolysis that led to clinical deterioration or an increase in the NIHSS score by more than 4 points in any intracranial location.

### Clinical outcomes

Long-term outcomes were assessed using mRS, with telephone follow-ups conducted at 90 days post-onset. Each patient’s mRS score was evaluated by two experienced neurologists without knowledge of the patients’ baseline data. An mRS score of 0–2 at 90 days was classified as a favorable functional outcome, while a score of 3–6 at 90 days was classified as an unfavorable functional outcome.

### Statistical analysis

All statistical analyses were performed using the Windows version of IBM SPSS Statistics 26 and GraphPad Prism 10.1.2 statistical package. Count data were presented as percentages, normally distributed data were presented as mean ± standard deviation, and non-normally distributed data were presented as median and interquartile range (IQR), i.e., the 25th to 75th percentile. Chi-square test was used to compare categorical variables among groups. For continuous variables, comparison of variance between groups was used for normally distributed data, and the Mann–Whitney U test was used for non-normally distributed data. According to the expected statistical analysis methods, the baseline characteristics of various types of AIS patients included in the study were firstly analyzed. Then the 90-day mRS scores and sICH were grouped and analyzed for their demographic and clinical characteristics. Predictive factors for the prognosis of each subtype of AIS were analyzed using binary logistic regression analysis, with Odds Ratio (OR) and 95% Confidence Interval (CI) obtained from univariate model regression analysis. Variables with a *p* value < 0.05 in the univariate model were selected as covariates for the multivariate model to obtain adjusted Odds Ratio (aOR) and 95%CI. Finally, receiver operating characteristic (ROC) curve analysis was conducted for variables demonstrating statistical significance (*p* < 0.05) in the multivariable logistic regression model, using the ROC curves to determine the Area Under Curve (AUC), sensitivity and specificity of the prediction, the best cutoff value for the ratio of inflammatory cells was determined using AUC and 95% confidence intervals, with an AUC of 0.70 or higher considered to indicate good predictive ability (18). A two-sided *p* < 0.05 was considered statistically significant.

## Results

### Baseline characteristics

**Figure 1:**
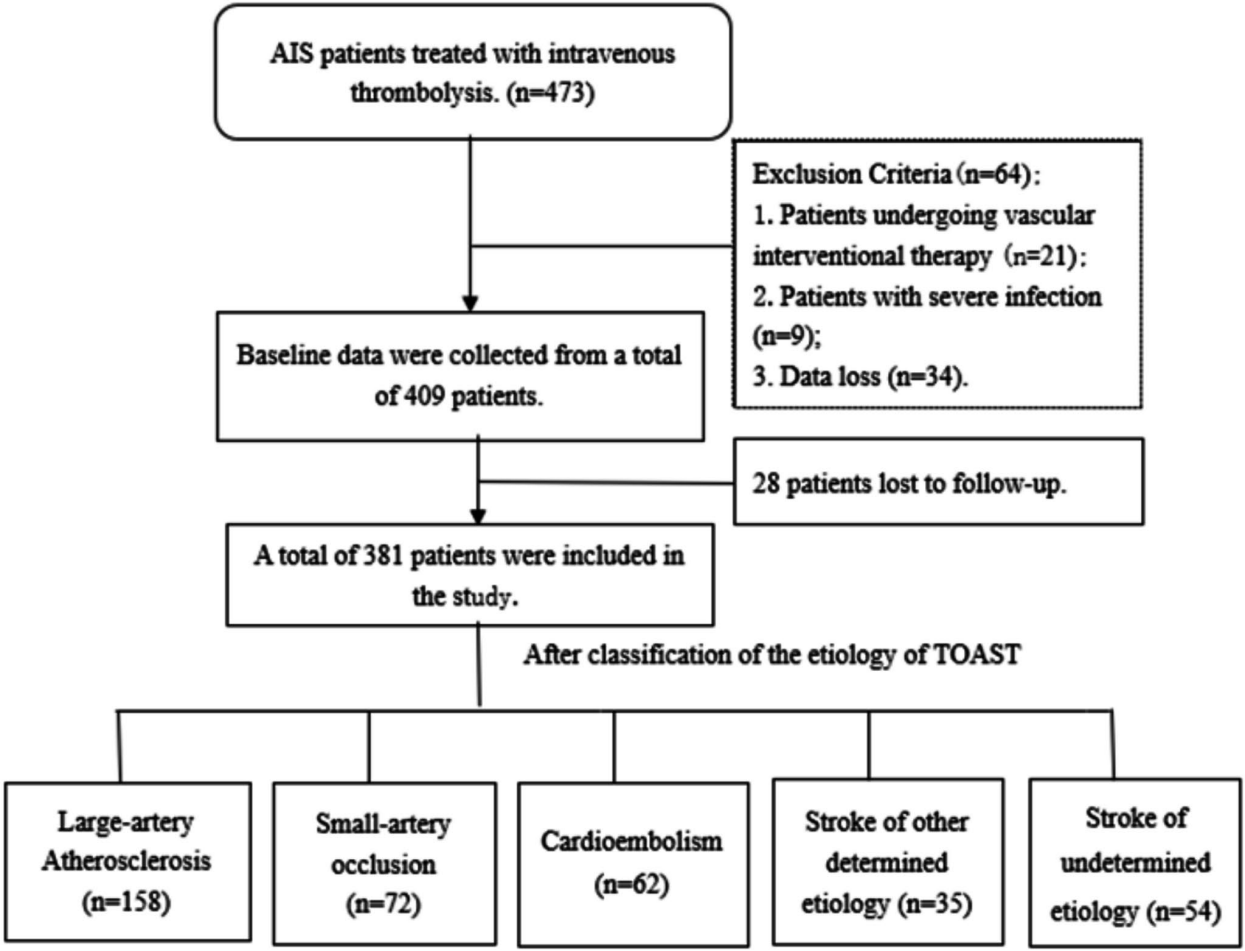
Flowchart of patient recruitment for inclusion in the study cohort.

From September 2018 to September 2023, of the 473 patients enrolled in our study, 34 (0.7%) patients were excluded because data were lost owing to update in the record system; 21 (4.4%) patients received endovascular intervention after thrombolytic therapy; 9 (1.9%) patients had severe infectious diseases before admission. Finally, 409 patients were included. At the follow-up period of 3 months, a total of 28 (5.9%) patients were lost to follow-up. Finally, 381 patients who met the criteria were included in this study (Figure 1).

Among the 381 included patients, the most common etiology was large-artery atherosclerosis (*n* = 158, 41.5%), followed by small-artery occlusion (*n* = 72, 18.9%), cardioembolism (*n* = 62, 16.3%), other determined etiology (*n* = 35, 9.2%), and undetermined etiology (*n* = 54, 14.2%). The demographic and clinical characteristics were compared according to TOAST classification (Table 1). The age was higher in cardioembolism, while younger in undetermined etiology (*p* < 0.001). The cardioembolism had higher proportions of history of atrial fibrillation (*p* < 0.001) and coronary heart disease (*p* = 0.021), and also had a higher NIHSS score (*p* < 0.001) on admission compared to other etiologies. Moreover, cardioembolism showed lower LMR values compared to other etiologies (*p* = 0.046). Regarding the location of infarction, large-artery atherosclerosis stroke was more likely to occur in the anterior circulation, while small-artery occlusion stroke was most commonly observed in the posterior circulation. The cardioembolism was more likely to lead to infarction involving both anterior and posterior circulations (*p* < 0.001).

**Table 1:** Baseline characteristics of AIS patients according to TOAST classification subtypes.

Variable	Large-artery atherosclerosis	Small-artery occlusion	Cardioembolism	Stroke of other determined etiology	Stroke of undetermined etiology	*p*-value
	(*n* = 158)	(*n* = 72)	(*n* = 62)	(*n* = 35)	(*n* = 54)	
Demography						
Male (%)	108 (68.4)	42 (58.3)	32 (51.6)	25 (71.4)	35 (64.8)	0.125
Age [Y, *M* (*P_25_, P_75_*)]	69.5 (58.8–78.0)	65.0 (54.0–74.8)	75.5 (66.0–80.0)	68.0 (53.0–75.0)	63.5 (51.8–72.3)	<0.001
Risk factors (%)						
Hypertension	104 (65.8)	43 (59.7)	40 (64.5)	19 (54.3)	29 (53.7)	0.443
Diabetes	39 (24.7)	23 (31.9)	13 (21.0)	6 (17.1)	9 (16.7)	0.252
Hyperlipidemia	40 (25.3)	22 (30.6)	15 (24.2)	8 (22.9)	19 (35.2)	0.551
Atrial fibrillation	11 (7.0)	0 (0.0)	48 (77.4)	0 (0.0)	1 (1.9)	<0.001
Coronary heart disease	24 (15.2)	5 (6.9)	12 (19.4)	5 (14.3)	1 (1.9)	0.021
Previous TIA	7 (4.4)	2 (2.8)	4 (6.5)	2 (5.7)	0 (0.0)	0.210
Previous stroke	35 (22.2)	12 (16.7)	12 (19.4)	7 (20.0)	7 (13.0)	0.634
Current smoking	55 (34.8)	21 (29.2)	14 (22.6)	13 (37.1)	22 (40.7)	0.240
SBP (mmHg, $\bar{x}\pm s$ )	143.7 ± 20.9	145.8 ± 17.0	145.7 ± 19.4	142.4 ± 17.7	147.4 ± 22.5	0.701
Admission NIHSSS [*M* (*P_25_, P_75_*)]	6.0 (3.0–10.0)	4.0 (2.3–6.0)	8.5 (4.8–14.0)	4.0 (3.0–7.0)	5.0 (3.0–8.0)	<0.001
OTT [h, *M* (*P_25_, P_75_*)]	2.8 (2.0–3.5)	2.8 (2.0–3.9)	3.0 (2.2–3.8)	3.0 (1.9–3.5)	3.0 (2.4–3.8)	0.411
OBD [h, *M* (*P_25_, P_75_*)]	2.0 (1.0–2.5)	2.0 (1.0–3.0)	1.8 (1.0–2.7)	1.5 (1.0–2.9)	2.9 (1.5–2.7)	0.297
Lab results						
Neutrophil counts [×10^9^/L, *M* (*P_25_, P_75_*)]	5.1 (3.8–6.7)	4.2 (3.4–6.1)	5.4 (3.9–7.3)	5.5 (4.1–7.3)	5.3 (3.4–7.5)	0.124
Lymphocyte counts [×10^9^/L, *M* (*P_25_, P_75_*)]	1.6 (1.2–2.3)	1.7 (1.2–2.2)	1.6 (1.1–2.0)	1.9 (1.5–2.4)	1.7 (1.1–2.3)	0.097
Monocyte counts [×10^9^/L, *M* (*P_25_, P_75_*)]	0.4 (0.4–0.6)	0.4 (0.3–0.6)	0.5 (0.3–0.6)	0.5 (0.3–0.7)	0.5 (0.4–0.6)	0.304
Platelet counts [×10^9^/L, *M* (*P_25_, P_75_*)]	209.0 (165.8–255.0)	210.0 (166.0–238.0)	191.0 (152.8–228.0)	206.0 (182.0–242.0)	192.5 (171.3–218.5)	0.171
NLR [*M* (*P_25_, P_75_*)]	3.0 (2.0–5.1)	2.6 (1.8–4.1)	3.3 (2.4–5.3)	2.9 (1.7–4.6)	3.1 (2.0–6.2)	0.241
LMR [*M* (*P_25_, P_75_*)]	3.9 (2.8–4.9)	3.6 (2.7–5.8)	3.4 (2.4–4.1)	4.4 (2.9–5.8)	3.6 (2.5–4.6)	0.046
PLR [*M* (*P_25_, P_75_*)]	437.9 (325.9–647.0)	447.3 (336.8–741.1)	398.1 (289.8–507.0)	394.1 (296.8–590.2)	404.5 (289.9–530.4)	0.101
APTT [s, *M* (*P_25_, P_75_*)]	34.4 (32.1–37.9)	34.9 (33.0–38.5)	33.9 (30.9–37.1)	34.1 (31.5–37.8)	35.5 (31.3–39.1)	0.379
PT [s, *M* (*P_25_, P_75_*)]	13.1 (12.6–13.8)	13.2 (12.4–13.8)	13.1 (12.7–13.8)	13.1 (12.6–13.9)	13.4 (12.5–13.8)	0.966
INR [*M* (*P_25_, P_75_*)]	1.0 (1.0–1.1)	1.0 (1.0–1.1)	1.0 (1.0–1.1)	1.0 (1.0–1.1)	1.0 (1.0–1.1)	0.901
Blood glucose [mmol/L, *M* (*P_25_, P_75_*)]	7.2 (6.2–9.3)	6.9 (5.9–8.9)	7.0 (6.3–7.7)	7.1 (6.0–9.6)	6.8 (6.0–8.1)	0.382
LDL (mmol/L, $\bar{\;x}\pm s$ )	2.8 ± 0.9	2.6 ± 0.8	2.6 ± 0.9	2.8 ± 1.0	2.7 ± 0.7	0.639
Hemorrhage transformation (%)	17 (10.8)	1 (1.4)	17 (27.4)	0 (0.0)	3 (5.6)	<0.001
SICH	6 (3.8)	0 (0.0)	9 (14.5)	0 (0.0)	1 (1.9)	
aSICH	11 (7.0)	1 (1.4)	8 (12.9)	0 (0.0)	2 (3.7)	
mRS score of 0–2 (%)	83 (52.5)	61 (84.7)	29 (46.8)	27 (77.1)	39 (72.2)	<0.001
mRS score of 3–6 (%)	75 (47.5)	11 (15.3)	33 (53.2)	8 (22.9)	15 (27.8)	<0.001
Death in hospital (%)	12 (7.6)	0 (0.0)	3 (4.8)	0 (0.0)	0 (0.0)	0.002
Infarction location (%)						<0.001
Anterior circulation	110 (69.6)	20 (27.8)	33 (53.2)	20 (57.1)	28 (51.9)	
Posterior circulation	44 (27.8)	51 (70.8)	12 (19.4)	7 (20.0)	22 (40.7)	
Anterior–posterior circulation	4 (2.5)	1 (1.4)	17 (27.4)	8 (22.9)	4 (7.4)	

In terms of clinical outcomes, large-artery atherosclerosis stroke (7.6%) had the highest proportion of in-hospital mortality, followed by cardioembolism (4.8%; *p* = 0.002). The cardioembolism was more likely to result in hemorrhagic transformation compared to other etiologies (*p* < 0.001). Among patients with favorable functional outcomes, the small-artery occlusion stroke had the highest proportion (84.7%; *p* < 0.001). The cardioembolism had the highest proportion of unfavorable functional outcomes (53.2%; *p* < 0.001).

### Lymphocyte-related ratios and sICH

The demographic and clinical characteristics were compared in positive sICH group and negative sICH group (Table 2). The patients with positive sICH had advanced age (*p* = 0.009) and a higher rate of history of atrial fibrillation (*p* < 0.001), and a higher baseline NIHSS score [14.0 (9.0–17.8) vs. 5.0 (3.0–9.0); *p* < 0.001]. Anterior circulation infarcts or anterior–posterior circulation infarcts were more likely to develop sICH compared to posterior circulation infarcts (*p* < 0.001). For etiologies, cardioembolism was more likely to develop sICH compared to other etiologies of stroke (*p* < 0.001). However, there was no statistical difference between lymphocyte-related ratios and sICH.

**Table 2:** Analysis of symptomatic intracerebral hemorrhage.

	SICH		
Variable	Positive	Negative	*p*-value
	(*n* = 16)	(*n* = 365)	
Demography			
Male (%)	8 (50.0)	234 (64.1)	0.251
Age [Y, *M* (*P_25_, P_75_*)]	74.5 (71.0–84)	68.0 (57.0–77.0)	0.009
Risk factors (%)			
Hypertension	9 (56.3)	226 (61.9)	0.648
Diabetes	5 (31.3)	85 (23.3)	0.665
Hyperlipidemia	4 (25.0)	100 (27.4)	0.833
Atrial fibrillation	9 (56.3)	51 (14.0)	<0.001
Coronary heart disease	2 (12.5)	45 (12.3)	0.984
Previous TIA	0 (0.0)	15 (4.1)	0.865
Previous stroke	3 (18.8)	70 (19.2)	0.966
Current smoking	1 (6.3)	124 (34.0)	0.021
SBP (mmHg, $\bar{x}\pm s$ )	153.5 ± 28.0	144.5 ± 19.4	0.219
Admission NIHSSS [*M* (*P_25_, P_75_*)]	14.0 (9.0–17.8)	5.0 (3.0–9.0)	0.001
OTT [h, *M*(*P_25_,P_75_*)]	2.3 (1.8–3.2)	3.0 (2.0–3.6)	0.168
Lab results			
Neutrophil counts [×10^9^/L, *M* (*P_25_, P_75_*)]	6.2 (4.1–7.3)	5.0 (3.7–6.7)	0.143
Lymphocyte counts [×10^9^/L, *M* (*P_25_, P_75_*)]	1.8 (1.1–2.2)	1.6 (1.2–2.2)	0.878
Monocyte counts [×10^9^/L, *M* (*P_25_, P_75_*)]	0.5 (0.3–0.6)	0.5 (0.3–0.6)	0.786
Platelet counts [×10^9^/L, *M* (*P_25_, P_75_*)]	170.5 (153.5–226.3)	203 (167.5–241.0)	0.116
NLR [*M* (*P_25_, P_75_*)]	3.2 (2.0–6.7)	3.0 (2.0–4.9)	0.526
LMR [*M* (*P_25_, P_75_*)]	4.1 (2.8–5.3)	3.7 (2.7–4.9)	0.592
PLR [*M* (*P_25_, P_75_*)]	389.9 (298.3–491.2)	430.1 (322.5–642.1)	0.426
APTT [s, *M* (*P_25_, P_75_*)]	34.0 (32.1–37.7)	34.6 (31.9–38.2)	0.944
PT [s, *M* (*P_25_, P_75_*)]	13.2 (12.6–13.6)	13.2 (12.6–13.8)	0.674
INR [*M* (*P_25_, P_75_*)]	1.0 (1.0–1.1)	1.0 (1.0–1.1)	0.393
Blood glucose [mmol/L, *M* (*P_25_, P_75_*)]	6.9 (5.9–7.9)	7.0 (6.1–8.8)	0.570
LDL (mmol/L, $\bar{x}\pm s$ )	2.7 ± 0.9	2.7 ± 0.9	0.863
Infarction location (%)			0.001
Anterior circulation	14 (87.5)	197 (54.0)	
Posterior circulation	0 (0.0)	136 (37.3)	
Anterior–posterior circulation	2 (12.5)	32 (8.8)	
TOAST classification (%)			<0.001
Large-artery Atherosclerosis	6 (37.5)	152 (41.6)	
Small-artery occlusion	0 (0.0)	72 (19.7)	
Cardioembolism	9 (56.3)	53 (14.5)	
Other determined etiology	0 (0.0)	35 (9.6)	
Undetermined etiology	1 (6.3)	53 (14.5)	

### 90-day clinical outcomes

**Figure 2:**
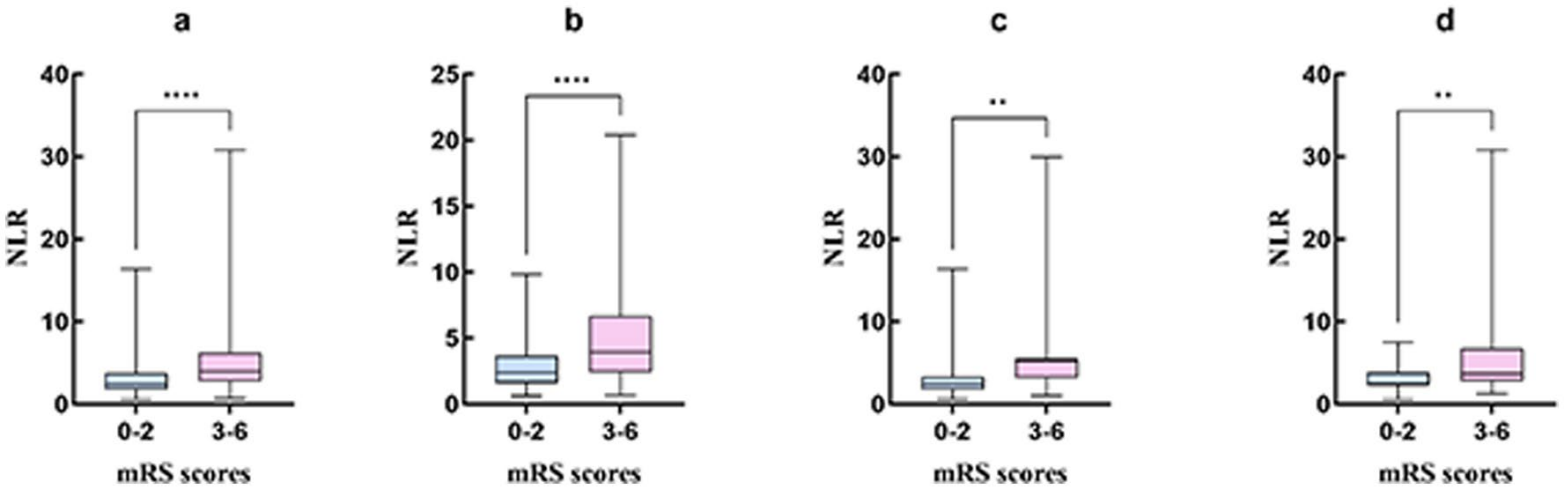
The graphical analysis of NLR and the 90-day mRS score of 3–6 for TOAST subtypes. **(A)** Included patients; **(B)** Large-artery atherosclerosis stroke; **(C)** Small-artery occlusion stroke; **(D)** Cardioembolic stroke. ^**^p < 0.01; ^****^p < 0.0001. ***indicates statistical significance at *p* < 0.001.

Comparison of the clinical characteristics and lymphocyte ratio between AIS patients with favorable functional outcome and those with unfavorable functional outcome at 90 days (Table 3). Male patients were more likely to have favorable functional outcome at 90 days after onset (*p* = 0.014). The patients in unfavorable functional outcome group were associated with advanced age (*p* < 0.001), a history of atrial fibrillation (*p* < 0.001), and higher NIHSS scores at admission [9.0 (6.0–14.0) vs. 4.0 (2.0–6.0); *p* < 0.001]. For the laboratory results, higher neutrophil counts [6.3 (4.6–8.5) vs. 4.5 (3.4–6.1); *p* < 0.001] and NLR [4.0 (2.7–6.3) vs. 2.4 (1.8–3.7); *p* < 0.001], lower lymphocyte counts [1.6 (1.1–2.1) vs. 1.7 (1.3–2.3); *p* = 0.005] and LMR [3.4 (2.2–4.5) vs. 3.8 (2.9–5.0); *p* < 0.001], and shorter APTT [33.5 (31.3–37.2) vs. 35.0 (33.2–38.6); *p* = 0.003] were more likely to result in unfavorable functional outcome at 90 days. Additionally, hemorrhage transformation (*p* < 0.001), anterior circulation stroke or anterior–posterior circulation stroke (*p* = 0.001), and large-artery atherosclerosis stroke or cardioembolic stroke (*p* < 0.001) were also more likely to result in an unfavorable functional outcome. In Figure 2, we graphically analyzed the influence of NLR on the 90-day mRS scores for TOAST subtypes, and found that higher NLR was associated with poor functional outcome patients with large-artery atherosclerosis stroke (*p* < 0.0001), small-artery occlusion stroke (*p* < 0.01), and cardioembolic stroke (*p* < 0.01).

**Table 3:** Analysis of mRS scores at 90 days.

	Neurological functional outcomes at 90 days		
Variable	mRS score of 0~2	mRS score of 3~6	*p*-value
	(*n* = 239)	(*n* = 142)	
Demography			
Male (%)	163 (68.2)	79 (55.6)	0.014
Age [Y, *M* (*P_25_, P_75_*)]	66.0 (54.0–75.0)	72.5 (61.8–80.0)	<0.001
Risk factors (%)			
Hypertension	142 (59.4)	93 (65.5)	0.238
Diabetes	58 (24.3)	32 (22.5)	0.700
Hyperlipidemia	67 (28.0)	37 (26.1)	0.675
Atrial fibrillation	20 (8.4)	40 (28.2)	<0.001
Coronary heart disease	32 (13.4)	15 (10.6)	0.417
Previous TIA	13 (5.4)	2 (1.4)	0.050
Previous stroke	40 (16.7)	33 (23.2)	0.119
Current smoking	87 (36.4)	38 (26.8)	0.053
SBP (mmHg, $\bar{x}\pm s$ )	143.6 ± 19.9	146.9 ± 19.9	0.119
Admission NIHSSS [*M* (*P_25_, P_75_*)]	4.0 (2.0–6.0)	9.0 (6.0–14.0)	<0.001
OTT [h, *M*(*P_25_,P_75_*)]	3.0 (2.0–3.6)	3.0 (2.2–3.5)	0.777
Lab results			
Neutrophil counts [×10^9^/L, *M* (*P_25_, P_75_*)]	4.5 (3.4–6.1)	6.3 (4.6–8.5)	<0.001
Lymphocyte counts [×10^9^/L, *M* (*P_25_, P_75_*)]	1.7 (1.3–2.3)	1.6 (1.1–2.1)	0.005
Monocyte counts [×10^9^/L, *M* (*P_25_, P_75_*)]	0.5 (0.3–0.6)	0.5 (0.4–0.6)	0.071
Platelet counts [×10^9^/L, *M* (*P_25_, P_75_*)]	201.0 (169.0–237.0)	155.0 (143.0–165.0)	0.795
NLR [*M* (*P_25_, P_75_*)]	2.4 (1.8–3.7)	4.0 (2.7–6.3)	<0.001
LMR [*M* (*P_25_, P_75_*)]	3.8 (2.9–5.0)	3.4 (2.2–4.5)	<0.001
PLR [*M* (*P_25_, P_75_*)]	434.1 (333.3–643.6)	398.1 (287.6–620.0)	0.162
APTT [s, *M* (*P_25_, P_75_*)]	35.0 (33.2–38.6)	33.5 (31.3–37.2)	0.003
PT [s, *M* (*P_25_, P_75_*)]	13.2 (12.6–13.8)	13.2 (12.6–13.8)	0.974
INR [*M* (*P_25_, P_75_*)]	1.0 (1.0–1.1)	1.0 (1.0–1.1)	0.702
Blood glucose [mmol/L, *M* (*P_25_, P_75_*)]	6.9 (6.0–8.6)	7.2 (6.1–8.9)	0.27
LDL (mmol/L, $\bar{x}\pm s$ )	2.7 ± 0.9	2.8 ± 0.9	0.215
Hemorrhage transformation (%)	11 (4.6)	27 (19.0)	<0.001
SICH	0 (0.0)	16 (11.3)	
aSICH	11 (4.6)	11 (7.7)	
Infarction location (%)			<0.001
Anterior circulation	122 (51.0)	89 (62.7)	
Posterior circulation	102 (42.7)	34 (23.9)	
Anterior–posterior circulation	15 (6.3)	19 (13.4)	
TOAST classification (%)			<0.001
Large-artery Atherosclerosis	83 (34.7)	75 (52.8)	
Small-artery occlusion	61 (25.5)	11 (7.7)	
Cardioembolism	29 (12.1)	33 (23.2)	
Other determined etiology	27 (11.3)	8 (5.6)	
Undetermined etiology	39 (16.3)	15 (10.6)	

### Univariable logistic regression analysis of 90-day clinical outcomes

Univariate logistic regression analysis indicated that male (OR, 0.585; 95%CI, 0.381–0.898; *p* = 0.014), age (OR, 1.032; 95%CI, 1.015–1.050; *p* < 0.001), history of atrial fibrillation (OR, 4.294; 95%CI, 2.390–7.715; *p* < 0.001), hemorrhage transformation (OR, 4.866; 95%CI, 2.331–10.159; *p* < 0.001), and baseline NIHSS score (OR, 1.291; 95%CI, 1.215–1.372; *p* < 0.001) were associated with adverse functional outcome at 90 days (Table 4). When using posterior circulation infarction as the reference, both anterior circulation infarction (OR, 2.189; 95%CI, 1.361–3.518; *p* = 0.001) and anterior–posterior circulation infarction (OR, 3.800; 95%CI, 1.741–8.292; *p* = 0.001) were associated with unfavorable functional outcome. In the TOAST classification, when using small-artery occlusion as the reference, large-artery atherosclerosis (OR, 5.011; 95%CI, 2.454–10.232; *p* < 0.001) and cardioembolic (OR, 6.310; 95%CI, 2.799–14.229; *p* < 0.001) strokes were associated with unfavorable functional outcome. Additionally, in the laboratory results, neutrophil count (OR, 1.413; 95%CI, 1.275–1.565; *p* < 0.001), lymphocyte count (OR, 0.684; 95%CI, 0.522–0.898; *p* = 0.006), monocyte count (OR, 3.423; 95%CI, 1.357–8.634; *p* = 0.009), NLR (OR, 1.217; 95%CI, 1.127–1.313; *p* < 0.001), and LMR (OR, 0.784; 95%CI, 0.691–0.890; *p* < 0.001) were associated with adverse functional outcome.

**Table 4:** Univariate analysis of characteristics of patients with AIS to identify predictors of 90-day unfavorable neurological outcome.

	mRS score of 3~6 at 90 days.		
Variable	OR	95%CI	*p*-value
Demography			
Male	0.585	0.381–0.898	0.014
Age (y)	1.032	1.015–1.050	<0.001
Risk factors			
Hypertension	1.296	0.842–1.996	0.238
Diabetes	0.908	0.555–1.485	0.700
Hyperlipidemia	0.905	0.566–1.446	0.675
Atrial fibrillation	4.294	2.390–7.715	<0.001
Coronary heart disease	0.764	0.398–1.466	0.418
Previous TIA	0.248	0.055–1.117	0.069
Previous stroke	1.506	0.898–2.525	0.12
Current smoking	0.638	0.405–1.007	0.053
SBP (mmHg)	1.008	0.998–1.019	0.119
Admission NIHSSS	1.291	1.215–1.372	<0.001
OTT (h)	1.020	0.830–1.254	0.853
Lab results			
Neutrophil counts (×10^9^/L)	1.413	1.275–1.565	<0.001
Lymphocyte counts (×10^9^/L)	0.684	0.522–0.898	0.006
Monocyte counts (×10^9^/L)	3.423	1.357–8.634	0.009
Platelet counts (×10^9^/L)	1.002	0.998–1.005	0.358
NLR	1.217	1.127–1.313	<0.001
LMR	0.784	0.691–0.890	<0.001
PLR	1.000	0.999–1.001	0.671
APTT (s)	0.984	0.954–1.016	0.323
PT (s)	1.096	0.983–1.223	0.098
INR	0.975	0.823–1.156	0.773
Blood glucose (mmol/L)	1.027	0.964–1.093	0.413
LDL (mmol/L)	1.163	0.916–1.477	0.215
Hemorrhage transformation	4.866	2.331–10.159	<0.001
Infarction location			
Anterior circulation	2.189	1.361–3.518	0.001
Posterior circulation	Reference	Reference	
Anterior–posterior circulation	3.800	1.741–8.292	0.001
TOAST classification			
Large-artery Atherosclerosis	5.011	2.454–10.232	<0.001
Small-artery occlusion	Reference	Reference	
Cardioembolism	6.310	2.799–14.229	<0.001
Other determined etiology	1.643	0.594–4.544	0.339
Undetermined etiology	2.133	0.889–5.120	0.090

### Multivariable logistic regression analysis of 90-day clinical outcomes

After adjusting for sex, age, history of atrial fibrillation, hemorrhage transformation, infarction location, and admission NIHSS score as confounding factors, the multivariable model (Table 5) suggested that NLR before thrombolysis (aOR, 1.261; 95% CI, 1.148–1.384; *p* < 0.001) and LMR (aOR, 0.735; 95% CI, 0.622–0.868; *p* < 0.001) were independent predictors of unfavorable functional outcome in AIS patients after intravenous thrombolysis treatment. Among them, NLR could be regarded as an independent predictor of poor functional outcome in patients with large-artery atherosclerotic stroke (aOR, 1.354; 95% CI, 1.142–1.606; *p* = 0.001), small-artery occlusion stroke (aOR, 1.505; 95% CI, 1.081–2.096; *p* = 0.015), and cardioembolic stroke (aOR, 1.871; 95% CI, 1.065–3.101; *p* = 0.028). LMR was only an independent predictor of unfavorable functional outcome in patients with large-artery atherosclerosis stroke (aOR, 0.693; 95% CI, 0.541–0.886; *p* = 0.004).

**Table 5:** Multivariable analysis was used to investigate the effects of NLR and LMR on the 90-day unfavorable neurological outcomes based on TOAST classification.

	NLR			LMR		
TOAST	aOR^*^	95%CI	*p*-value	aOR^*^	95%CI	*p*-value
All included patients	1.261	1.148–1.384	<0.001	0.735	0.622–0.868	<0.001
Large-artery atherosclerosis	1.354	1.142–1.606	0.001	0.693	0.541–0.886	0.004
Small-artery occlusion	1.505	1.081–2.096	0.015	0.641	0.373–1.101	0.107
Cardioembolism	1.817	1.065–3.101	0.028	0.700	0.359–1.365	0.296
Other determined etiology	1.047	0.738–1.486	0.796	0.939	0.490–1.799	0.849
Undetermined etiology	1.053	0.857–1.293	0.623	0.736	0.395–1.373	0.335

^*^Adjusted for gender, age, history of atrial fibrillation, admission NIHSS score, location of infarct and hemorrhage transformation.

### Predictive ability of NLR and LMR

**Figure 3:**
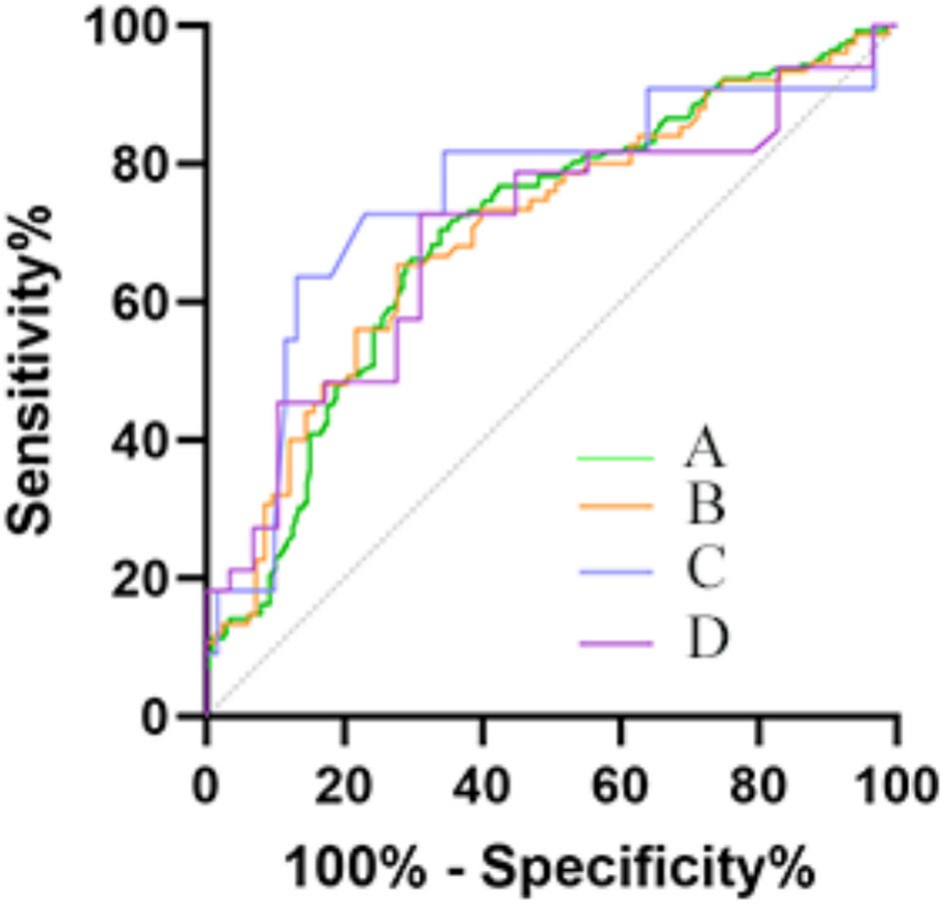
ROC curves were depicted for significant predictive value of mRS score 0f 3–6. **(A)** Included patients (Sensitivity: 0.662; Specificity: 0.707; Cutoff: 3.281); **(B)** Large-artery atherosclerosis subtype (Sensitivity: 0.653; Specificity: 0.733; Cutoff: 3.193); **(C)** Small-artery occlusion subtype (Sensitivity: 0.727; Specificity: 0.787; Cutoff: 3.937); **(D)** Cardioembolic subtype (Sensitivity: 0.727; Specificity: 0.690; Cutoff: 3.172).

The abilities of NLR and LMR to predict 90-day unfavorable functional outcome according to TOAST subtypes are summarized in Table 6, and presented the ROC curves with good results in Figure 3. The ROC values of NLR and LMR were well differentiated among the TOAST subtypes. Table 6 and Figure 3 indicated that the AUC values of NLR for all AIS patients, large-artery atherosclerosis stroke, small-artery occlusion stroke and cardioembolic stroke were 0.701, 0.702, 0.750, and 0.679 respectively, the sensitivities were 0.662, 0.653, 0.727 and 0.727 respectively, the specificities were 0.707, 0.733, 0.787 and 0.690, respectively. The cutoff values of NLR for predicting unfavorable functional outcome in all AIS patients, large-artery atherosclerosis stroke, small-artery occlusion stroke and cardioembolic stroke were 3.281, 3.193, 3.937 and 3.172, respectively. LMR had poor predictive ability for unfavorable functional outcome in large-artery atherosclerosis stroke with an AUC of 0.591, and its predictive ability for other subtypes were not statistically significant.

**Table 6:** ROC curves were used to analyze the prognostic value of unfavorable neurological outcomes at 90 days.

	NLR			LMR		
TOAST	AUC	95%CI	*p*-value	AUC	95%CI	*p*-value
All included patients	0.701	0.646–0.755	<0.001	0.615	0.555–0.674	<0.001
Large-artery Atherosclerosis	0.702	0.620–0.784	<0.001	0.591	0.502–0.679	0.049
Small-artery occlusion	0.750	0.572–0.928	0.009	0.656	0.463–0.850	0.100
Cardioembolism	0.679	0.565–0.829	0.008	0.606	0.464–0.748	0.152
Other determined etiology	0.685	0.508–0.862	0.116	0.708	0.482–0.934	0.077
Undetermined etiology	0.667	0.515–0.818	0.060	0.639	0.451–0.827	0.116

## Discussion

Our study involving 381 AIS patients underwent intravenous thrombolysis, there was significant differences in the clinical characteristics of AIS patients receiving intravenous thrombolysis based on TOAST classification. Admission higher NLR and lower LMR as peripheral inflammatory markers were associated with unfavorable functional outcomes at 90 days, but not with sICH. Otherwise, based on TOAST classification, NLR was associated with unfavorable functional outcomes in large-artery atherosclerosis stroke, small-artery occlusion stroke, and cardioembolic stroke, while LMR was only associated with unfavorable functional outcome in large-artery atherosclerosis stroke. The accuracy of NLR in predicting unfavorable functional outcomes in large-artery atherosclerotic stroke and small-artery occlusive stroke were good, but its accuracy in predicting prognosis for other etiologies were poor.

Previous studies have shown that a high NLR combined with a low LMR measured 24 h after thrombolysis was related with poor functional outcome at 90 days in AIS patients (19). A recent meta-analysis also suggested that a higher NLR was associated with poor functional outcome at 3 months, however, the time at which NLR was obtained was not specified before or after thrombolysis (15). Another large retrospective study of patients undergoing mechanical thrombectomy showed that the admission NLR was associated with 3-month mortality and sICH in patients with large-artery atherosclerosis stroke (20). Within 30 min of onset, the inflammation began to respond and neutrophils increased in circulating (9). A large prospective study suggested that elevated levels of plasma neutrophil elastase obtained upon admission were associated with adverse neurological outcomes at 3 months after receiving intravenous thrombolysis for AIS, which was released by neutrophils (21). Intravenous thrombolysis therapy could partially restore blood flow in ischemic penumbra and minimized brain tissue damage, while the immune system may still maintain an inflammatory response after vascular recanalization (22). The interaction between thrombolysis and inflammatory mechanisms drove the progression of neural damage in AIS (23). These lymphocyte-related ratios obtained before or after thrombolysis may be associated with adverse functional outcomes, and differed in their relationship with different etiologies of stroke. In our study, the baseline characteristics and prognostic factors of AIS with different etiologies showed significant differences. Rapid evaluation of prognosis of various etiological AIS before intravenous thrombolysis can provide clinical decision-making guidance.

Neutrophils played a role in releasing cytokines and inflammatory mediators, infiltrating and releasing oxygen free radicals, expressing elastase to disrupt the blood–brain barrier, and causing capillary stasis and neuronal ischemia and hypoxia, which resulted in neurological deterioration (11, 24, 25). Animal models of stroke suggested that the depletion of γδT cells had a damaging role post-stroke, meanwhile, CD8 + T cells aggregated in the necrotic area mediating cytotoxic reactions to disrupt the blood–brain barrier (11). Monocytes increased within hours after infarction, releasing deleterious inflammatory mediators that caused inflammation in the infarct area and promoted platelet-monocyte aggregate formation, which lead to thrombus formation and vascular occlusion (11, 26).

Chronic inflammation of the arterial wall also played a key role in the occurrence and development of atherosclerosis (27). The plaque rupture was caused by infiltration of neutrophils and increased neutrophil–platelet adhesion. Animal studies have shown that neutrophils infiltrated into arterial plaques, leading to chronic inflammation; additionally, neutrophils may also damage plaques by releasing proteolytic enzymes. Even within the normal range of white blood cell count, a higher NLR was still associated with atherosclerotic events (27). This may be one of the mechanisms by which a higher admission NLR can predict unfavorable neurological outcome at 90 days in large-artery atherosclerosis stroke and small-artery occlusion stroke. Moreover, high admission NLR has been shown to have predictive value for cerebral infarction progression during hospitalization in large-artery atherosclerosis stroke (28). In cardioembolic stroke, although high NLR was an independent predictor for adverse neurological outcome at 90 days post onset, its predictive value was limited. Other studies have suggested that higher NLR within 24 h of admission was predictive of the hemorrhage transformation in patients with atrial fibrillation-related AIS, and the relationship with poor neurological outcomes remained to be further confirmed (29).

In large-artery atherosclerotic stroke, exposure of the lipid-rich necrotic core within unstable plaques triggered T-lymphocyte infiltration, while systemic inflammation exacerbated lymphocyte apoptosis, leading to a reduction in peripheral blood lymphocyte absolute counts and consequently elevating the NLR (11, 27). This pathophysiological cascade justified the higher NLR cutoff observed in large-artery atherosclerotic stroke patients. In cardioembolic stroke, tissue factors released from cardiac thrombi or injured myocardium activated thromboinflammatory cascades (11), the absence of chronic arterial inflammation resulted in a milder decline in lymphocytes and thus a lower NLR cutoff compared to large-artery atherosclerotic stroke. In small-artery occlusion stroke, chronic blood–brain barrier leakage promoted peripheral lymphocyte migration into brain parenchyma (11), which may stabilize peripheral lymphocyte counts despite localized neuroinflammation, this unique mechanism likely contributed to the distinctively higher NLR cutoff compared to other subtypes.

Although low LMR obtained within 24 h of admission was associated hemorrhage transformation in AIS patients (30). NLR, LMR, and PLR at admission were not found to be related to sICH in AIS patients after receiving intravenous thrombolysis treatment in our study, and similar findings had been reported in other studies (31).

Furthermore, platelet-monocyte activation in stroke patients was significantly higher than in healthy individuals, leading to spontaneous platelet aggregation. After stroke onset, platelets were activated and aggregated, which caused neuronal damage (22, 32). In cardioembolic stroke, platelets played a pivotal role in the pathophysiological processes of cerebral infarction through activation of the coagulation cascade and microcirculatory obstruction. These mechanisms exacerbated inflammatory responses and propagated thrombus formation, thereby hindering stroke recovery and contributing to adverse clinical outcomes (33), which may result higher PLR obtained before thrombolysis has been found to be associated with early neurological deterioration in patients with AIS (13). However, in our study, we did not find evidence that pre-thrombolysis PLR was associated with sICH or poor neurological outcomes. This may be related with the lack of significant early changes in platelets, and similar findings have been reported in other studies (34). The limited predictive utility of LMR and PLR may relate to monocyte/platelet dynamics being more influenced by acute-phase responses rather than chronic vascular inflammation. Future studies should evaluate serial measurements of these ratios in conjunction with advanced imaging biomarkers to clarify their pathophysiological roles.

There were several limitations to this study. Firstly, it was a study with a relatively small sample size from a single center, the predictive performance for large-artery atherosclerotic stroke marginally exceeded the predefined threshold (AUC = 0.70), suggesting suboptimal discriminative capacity. Especially with fewer cases in other determined etiological stroke, there were only 35 cases were included in the multiple regression analysis, which substantially diminishes the statistical power to detect outcomes within this subtype. Thus, multivariate regression models analyzing this subgroup may yield imprecise OR with disproportionately wide CI, reducing the reliability of predictive inferences. While this limitation inherently reflected fundamental challenges in current etiological research on AIS, it simultaneously delineates actionable pathways for future investigations. Furthermore, establishing multicenter collaborative cohorts may prove particularly valuable for augmenting statistical power while preserving clinical heterogeneity across diverse populations. Importantly, our multivariate models did not adjust for post-thrombolysis interventions (e.g., antiplatelet timing) and complications (e.g., infections), which may confound inflammatory marker trajectories. While our cohort followed institutional protocols for standard care, residual confounding from unmeasured clinical decisions cannot be excluded. Future prospective studies should integrate real-time monitoring of both baseline and in-hospital variables to disentangle these effects. Secondly, we obtained lymphocyte-related ratios at admission, which was a single time point and restricted the evaluation of dynamic changes in inflammation after thrombolysis. Our reliance on baseline NLR/LMR precluded conclusions about the role of post-thrombolysis inflammatory dynamics. Prior studies had suggested that higher NLR within 24 h of admission was predictive of the hemorrhage transformation in patients with atrial fibrillation-related AIS, and the relationship with poor neurological outcomes remained to be further confirmed (29). Future prospective studies should integrate serial biomarker measurements to disentangle acute vs. secondary inflammatory effects on stroke outcomes. Finally, there was limited research on the relationship between NLR at admission and sICH after receiving intravenous thrombolysis for AIS. Furthermore, the observed AUC differences should be interpreted cautiously due to sample heterogeneity. Future studies with larger, overlapping cohorts are needed to rigorously validate subtype-specific performance differences.

Our study provided the first evidence on the prognostic utility of pre-thrombolysis lymphocyte-related ratios (e.g., NLR) in AIS patients with distinct etiological subtypes following intravenous thrombolysis. By focusing on pre-intervention biomarkers, we isolated early inflammatory responses inherent to the index stroke, thereby minimizing confounding effects from post-stroke complications (e.g., hospital-acquired infections) or secondary systemic inflammation. Notably, admission NLR emerged as an etiology-specific prognostic indicator, with validated cutoffs offering clinical utility for risk stratification and personalized therapeutic decision-making. Further investigations should elucidate the pathophysiological interplay between pre-thrombolysis inflammatory profiles and stroke subtypes, while expanding biomarker panels to include cytokines and neutrophil elastase for precise outcome prediction.

## Conclusion

NLR had a borderline adequate predictive capacity for adverse neurological outcomes in large-artery atherosclerosis stroke and a reasonable predictive performance for small-artery occlusion stroke, the NLR may serve as an important prognostic marker for AIS, assisting in evaluating therapeutic efficacy and selecting treatment modalities in clinical decision-making through cut-off values.

## Data Availability

The raw data supporting the conclusions of this article will be made available by the authors, without undue reservation.

## Glossary

AIS: Acute ischemic stroke
aOR: Adjusted odds ratio
APTT: Activated partial thromboplastin time
aSICH: Asymptomatic intracerebral hemorrhage
AUC: Area under the receiver operating characteristic curve
CI: Confidence interval
INR: International normalized ratio
LDL: Low density lipoprotein
LMR: Lymphocyte to monocyte ratio
M: Median
mRS: modified Rankin Scale
NIHSS: National Institutes of Health Stroke Scale
NLR: Neutrophil to lymphocyte ratio
ODB: Onset to draw blood
OR: Odds ratio
OTT: Onset to thrombolysis time
PT: Prothrombin time
PLR: Platelet to lymphocyte ratio
ROC: Receiver operating characteristic curve
SICH: Symptomatic intracerebral hemorrhage
TOAST: Trial of Org 10,172 in Acute Stroke Treatment
TIA: Transient ischemic attack
Y: Years old
